# Shall We Discontinue Devitalization?

**Published:** 1916-06

**Authors:** Walter S. Kyes


### SHALL WE DISCONTINUE DEVITALIZATION?

BY WALTER S. KYES, D.D.S.

I want to discuss briefly the substance of a paper read by Henry L. Ulrich, M.D., before the Minnesota Academy of Medicine, and later published in the *Journal-Lancet,* November, 1915.

I have no hope or desire to add to or detract from the value of the paper; neither shall I endeavor to corroborate nor contradict any of the statements made therein, my object in discussing it being none other than to get the matter before the readers of the DIGEST and making such comments as my experience and observation as a practising dentist have taught me.

The paper is entitled "Streptococcicosis." The author, taking all responsibility for the terminology and giving a number of reasons for so doing.

The two main factors which the writer states he wishes to bring out in the study are, "The diversity of clinical manifestations of 'streptococcal focal diseases,' and 'The significance of the blind apical abscess in' streptococcal focal diseases."

Speaking of the doctrine of focal infections the writer states that, "It is the tremendous extent of distribution, its adaptability to a variety of foci, its protean clinical manifestations, which have given the streptococcus this important distinction. "Another very important fact co-existing with this doctrine is the splendid demonstration of the laws of mutation virulence, and selective actions which these mutants exercise in establishing foci.

It would seem that these foci of infection are established without regard for race, color or location, being found in all the organs more or less, including the tonsils, ulcers of the stomach and the appendix and "last but not least in importance, the blind abscesses at the roots of devitalized teeth."

"The root abscess," continues the writer, "is far more important, far more significant, than has heretofore been realized. If it were possible to tabulate all the foci outside the respiratory tract, or rather outside the tonsils, the root abscesses of devitalized teeth would lead the list by a large majority."

"Fifty cases in which blind apical abscesses were present, the cultures of which abscesses gave types of streptococci, were analysed as to age, sex and clinical findings."

The author groups these fifty cases into five groups and two sub-groups, three of which I will use for illustration.

1. Rheumatoid group (24 cases).

2. Cardiovascular group (5 cases).
3. Asthenic group (11 cases).

Speaking of the clinical pathogenicity in group 1 the author states, "There were several instances in which no results were obtained by removal of the tonsils alone. Not until the dental lesions were destroyed was there prompt restitution. Other cases reported the removal of the tonsils and all other foci except the mouth foci. The reduction of these again gave quick response. The use of vaccines prepared from bacteria prepared from apical lesions, gave focal reactions, which is abundant proof of specificity. In some instances the removal of some of the foci of the mouth gave partial relief. On removal of all foci in the mouth, including teeth of a suspicious appearance, complete and permanent results were obtained."

In discussing group 2 the author states that, "The proliferation action of streptococci on vascular endothelium may well give rise to a form of endarteritis resulting in general sclerosis with or without subsequent hypertension."

I quote also the following in regard to group 2, "The value of the skiagraph of the mouth in establishing an additional or remaining depot of focal infection in streptococcal disease is unquestioned. The apical abscess may be the only focus left, the evacuation of which will permit of the reestablishment of renewed integrity of all parts.

"It may hold the balance of power in the struggle of the body for complete sterilization."

The writer then quotes some figures founded on his investigations which are both interesting and appalling to the dental profession.

After examining the röntgenographic films of 387 cases in which 997 teeth came under suspicion, he deducts the following: "By conservative interpretation, 737 root abscesses were seen. There were present 806 artificially devitalized teeth, of these 545 had blind abscesses at the tip of the roots, and 191 abscesses were present on teeth devitalized either by accident or pulp destruction by caries."

About the only consolatory feature of these figures is, that the dental profession was not apparently responsible for the 191 abscesses that resulted from caries and accident.

It occurs to me that it would require further study and a closer analysis of the cases examined to prove that our best efforts in the matter of root canal filling are wrought with such calamitous failure and with such possible deleterious effects on the health of our clientele.

It is a well known fact that certain practitioners follow out with varying degrees of effort and sincerity, certain methods of pulp devitalization and root canal treatment.

For instance, we have those who adhere to the chloro-percha and gutta percha point method of root canal filling under the protection of the rubber dam and various antiseptic agents; again we have those operators whose proud boast is that they have not had a roll of rubber dam in their office for a number of years and have treated teeth continuously.

There are also a vast number of operators who use the various mummifying agents, with and without the use of the rubber dam.

The pressure anesthesia method of pulp removal has a host of adherents who are more or less skilled in the removal of pulp débris without the rubber dam.

Others are as equally enthusiastic about the use of medicated pastes and mixtures of medicinal agents for root canal fillings.

The writer knows of several offices where hundreds of teeth are treated every month and no attempt whatever is made to remove the pulp or to fill the root canals.

The question that presents itself is, what method of treatment and root canal filling was made use of in the 737 root abscesses observed by Dr. Ulrich?

It is highly probable that this information could not be obtained from cases selected at random, but the information would be available if the cases of a single operator using a particular method were observed, and in this manner the best method could be arrived at, providing such a one exists.

Further elucidating the author continues, "The prevention of such abscesses entails a new dental attitude. It is my *impression* that in the days when dentists removed pulpless teeth, the statistics of chronic rheumatoid conditions were much lower than today."

The only way in which this impression could be verified would be to produce the rheumatoid and dental statistics together with those covering the increase in population for a certain period which is in all probability more difficult than to fill correctly, a root canal.

"Chronic rheumatoid conditions are decidedly on the increase and I attribute it to the prevalent custom of dentists to save teeth," continues the writer. If this attribution proves true it will necessarily revise the teachings of both medicine and dentistry in the matter of the preservation of the teeth.

After stating that the apical abscess in his opinion is hematogenous in origin, and that "The devitalization of teeth, which entails the destruction of nerve and blood supply to the apex and continguous bone areas, produces a locus resistentiae minoris, with lowered oxygen pressure, thereby creating an ideal nidus for streptococcal growth. We are compelled by the logic of the situation to condemn all efforts at devitalization by dentists, and strongly to urge extraction of teeth which need removal of pulp."

He then cites the findings of Drs. Best and Davis who oppose this view, and who collected 135 cases where the root canal fillings were done by "other dentists." They reported 128 defective root canal fillings with 103 abscesses. It is perhaps needless to here remark that had these investigative gentlemen collected cases from among their own clientele, their contribution to dentistry would have probably been of much more value.

We would have at least known the method of root canal filling employed and might have been a little nearer the solution of the problem. As it is, their investigations only go to prove that the correct filling of a root canal by "other dentists" is merely an accident and that 25 out of 128 defective root canal fillings were not abscessed, which should at least lead us to believe that not all our efforts along this line result in failure.

There is no questioning the fact that Dr. Ulrich has thrown a bomb into the quietude and prosperity of our professional citadel which calls for some very thorough and painstaking investigation on our part, the ultimate outcome of which will be watched with deep interest by the medical as well as the dental profession.—*Dental Digest,* for April.

